# Reliability of the Cunningham Panel

**DOI:** 10.1038/s41398-019-0462-1

**Published:** 2019-04-08

**Authors:** Richard E. Frye, Craig Shimasaki

**Affiliations:** 1 Barrow Neurological Institute at Phoenix Children’s Hospital, Phoenix, AZ USA; 2 Department of Child Health, University of Arizona College of Medicine, Phoenix, AZ USA; 3 Moleculera Labs, Inc, Oklahoma City, OK USA

The authors of Connery et al.^1^ thank Bejerot and Hesselmark^2^ for opening the discussion of the reliable and valid use of the Cunningham Panel as it raised misconceptions that we are eager to address.

The Connery et al.^1^ study treated patients that had both autoantibody elevation, and elevated CaMKII values. The clinical treatment protocol utilized this approach to ensure that the most appropriate patients were treated and those less likely to respond to immunotherapy were not exposed to inappropriate treatment. The clinical value of a medical test is highly dependent on applying it to an appropriate symptomatic disease population. Thus, applying the test to a subset of individuals who are unlikely to demonstrate a response to treatment, as suggested by Bejerot and Hesselmark^2^, would indeed change its accuracy and clinical utility but only because the test was applied inappropriately. It must be remembered that the Cunningham Panel, like other medical tests, should not be applied in isolation, and the panel is currently used as an aid in diagnosis, rather than as a substitute for careful clinical evaluation.

Bejerot and Hesselmark^2^ criticize the Connery et al.^1^ study based on a previous report^3^ of a retesting study of 53 patients with 46 patients having a repeat test panel performed. However, Bejerot and Hesselmark^2^ neglected to mention that they utilized invalid blood collection tubes containing excipients which render the specimens void in the study they reference to support their claims. A Corrigendum to their study^4^ stated *“The use of a blood collection tube other than the one recommended by Moleculera could be viewed as a limitation to our study.”* The authors acknowledged the critical tube collection study flaw, but somehow maintain that their results are still reliable. Invalid blood collection methods alone would render the results questionable at best, but potential assay interfering substances which could alter the results would render the study void.

An additional flaw in the Hesselmark and Bejerot^3^ study is their inability to provide representative healthy controls. Many of their “healthy controls” demonstrated an elevation in the antibody-mediated cell signaling calcium calmodulin-dependent protein kinase II (CaMKII) activation assay. The reasons for elevated autoantibodies in the ELISA or CaMKII in the “healthy controls” used in the Hesselmark and Bejerot study are likely due to (1) inclusion of a mixed age range (adults and children), (2) invalid collection methods, and (3) insufficient exclusion and inclusion criteria. Family history of psychiatric, autoimmune, or movement disorder was not an exclusion criterion for their controls. Patients who had psychiatric care greater than one year prior to enrollment appear to have been included as healthy controls based upon their stated exclusion criteria. There was no indication that they excluded or screened for recent or active infections. It is well-known that autoantibodies can be elevated for months to years preceding the development of symptoms of autoimmune disease^5^, and that normal unaffected populations can have autoantibodies due to infections and/or microbial antigen cross-reactivity^6,7^. Thus, the presence of autoantibodies found in the healthy volunteers emphasizes the need for careful selection of controls.

**Fig. 1:**
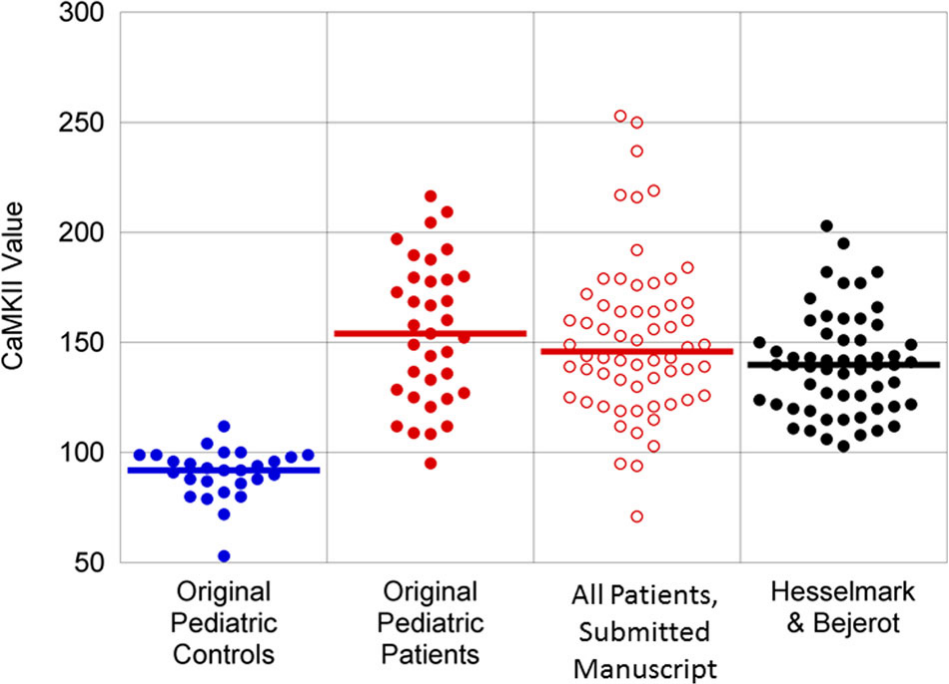
CaMKII results in PANDAS/PANS patients by group. “Original Pediatric Controls” and “Original Pediatric Patients” are the populations originally used to define the threshold of positivity (130) for the CaMKII assay^6^ (Kirvan, C.A., et al., 2006). “All Patients, submitted manuscript” are the values for the CaMKII assay for all patients in a manuscript now under review. “Hesselmark & Bejerot” are all patients as described in ref. ^3^. Performance metrics can vary in different studies based on the diseased population selected, the inclusion and exclusion criteria for the control population, the impact of interfering substances, and the specimen handling methods in ref. ^4^

When we compared CaMKII values from pediatric acute-onset neuropsychiatric syndrome (PANS) patients in the Hesselmark and Bejerot^3^ study to CaMKII values of PANS patients in our previously published studies, we found that they were comparable to our PANS patient CaMKII scores and were appropriately discriminated from our original pediatric controls from Yale University and the National Institute of Mental Health, USA, which have been established for the Cunningham Panel. Thus, using our established pediatric controls, we found clear differences between the CaMKII values from their PANS cases and our healthy controls (Fig. 1).

In the Connery et al.^1^ study, the Cunningham Panel predicted patients’ response to IVIG treatment with a sensitivity of 90–100%, a specificity of 67–75%, and an overall accuracy between 81 and 88%. The fact that patients responded to immunotherapy based upon a panel that identifies elevated antineuronal antibodies against the basal ganglia, emphasizes the importance of determining an underlying etiology or co-morbidity prior to treatment. We believe that the misguided conclusions of Bejerot and Hesselmark^2^ are based on inappropriate healthy controls and/or improper sample collection in the Hesselmark and Bejerot^3^ study.
